# Tetrasomy 18p Initially Misdiagnosed as Cerebral Palsy in an Adult Patient

**DOI:** 10.7759/cureus.20053

**Published:** 2021-11-30

**Authors:** Yusuf Mehkri, Rebecca Jules, Aisha Elfasi, Hans Shuhaiber

**Affiliations:** 1 Neurosurgery, University of Florida, Gainesville, USA; 2 Neurology, University of California, Los Angeles, USA; 3 Neurology, University of Florida, Gainesville, USA

**Keywords:** cerebral palsy, multisystem, chromosome 18, genetics, tetrasomy 18p syndrome

## Abstract

Tetrasomy 18p is a rare genetic condition characterized by a supernumerary 18p isochromosome with two copies of the p arm of chromosome 18 causing patients to have an extra chromosome. Most cases are de novo; however, a few maternally inherited cases have been reported. The most commonly reported manifestations of this condition are developmental delay, cognitive impairments, muscle tone abnormalities, and dysmorphic facial features. This case details a new diagnosis of tetrasomy 18p in a 42-year-old adult who was initially diagnosed with cerebral palsy as a child. We compare the phenotypic traits of our patient with the ones reported in the literature.

## Introduction

Tetrasomy 18p is a rare genetic condition in which there is a supernumerary 18p isochromosome with two copies of the p arm of chromosome 18; thus, patients with this condition have 47 chromosomes instead of the usual 46 [1]. Tetrasomy 18p is found equally in males and females, affecting about one in 140,000 live births [1,2]. While the majority of cases are de novo, there are a few maternally inherited cases [1,3].

The tetrasomy 18p syndrome has been associated with several mental and physical impairments spanning across several organ systems. According to a review of 43 patients with this condition, Sebold et al. described the main characteristic findings of patients with tetrasomy 18p as growth retardation, microcephaly, strabismus, abnormalities in muscle tone, and scoliosis/kyphosis. Other notable features included neonatal jaundice, recurrent otitis media, hearing loss, seizures, refractive errors, a history of constipation and gastroesophageal reflux, heart defects, and pes planus [4]. Various types of magnetic resonance imaging (MRI) abnormalities were noted on imaging, including enlargement of the lateral ventricles, thin or small corpus callosum, and minor signal abnormalities. Other less commonly reported features included kidney defects, hernias, and myelomeningocele, as well as short stature with failure to respond to growth hormone stimulation testing. The features most commonly reported during genetics evaluation included a smooth philtrum, small/malformed ears, palatal abnormalities, and camptodactyly/finger contractures. The diagnosis of tetrasomy 18p syndrome is achieved through karyotyping or fluorescence in situ hybridization (FISH) by centromere-specific probes or comparative genomic hybridization microarray [4,5]. Due to the many systems affected by this syndrome, patients with tetrasomy 18p require extensive medical workup in order to diagnose the manifestations of this disease. Patients require a multidisciplinary approach to their care comprising full genetic counseling and evaluation; neurological evaluation for seizures and magnetic resonance imaging to evaluate for brain abnormalities; gastrointestinal evaluations for constipation, reflux, and failure to thrive; orthopedic evaluation for kyphosis/scoliosis; genitourinary evaluation for renal defects; and occupational and physical therapy to aid in independence [6]. As of now, there is no cure for this syndrome, and the available literature on this condition is scarce.

In this paper, we describe the phenotypic features of a 42-year-old male newly diagnosed with tetrasomy 18p syndrome and compare those findings to those found in the literature.

## Case presentation

Clinical features

The patient is a 42-year-old male who was referred to the University of Florida Neurology Department for evaluation of mental retardation. He was born to a single, teenage mother and believed to be full term. The mother did not have prenatal care, and the patient was born with low birth weight. However, the actual weight at birth is unknown. Additional newborn history is also unknown, although he was diagnosed with cerebral palsy and scoliosis as a child.

Recently, he was diagnosed with hypertension and chronic kidney disease stage III, likely secondary to acute kidney injury from *Escherichia coli* sepsis about four years ago.

He currently lives with his sister and her husband and requires assistance with activities of daily living. He has completed up to grade school. He is able to perform some activities of daily living such as making his bed and helping with household chores. He helps tend the pets at home. He has difficulty with short-term memory. He is able to write his name; however, he cannot read. He has also had some behavioral issues; he occasionally becomes angry.

On examination, he was noted to have clinical features including intellectual disability (low IQ score reported by family), a towerlike head appearance, and excessive smiling. He was also noted to have excessive skin pads at the tips of his fingers, bilateral clinodactyly, dry hand palms, normal set ears, and ear cupping. Gait difficulties were also present.

Due to concerns for a genetic syndrome, he underwent a complete genetic evaluation with magnetic resonance imaging of the brain and an echocardiogram. The results of those studies are listed below.

Chromosomal and molecular analysis

Cytogenic testing and chromosome analysis demonstrated the following: an unbalanced male karyotype and containing an isochromosome 18 short arm accompanied by two normal copies of chromosome 18 homolog. The resulting karyotype is tetrasomic for the short arm of chromosome 18. The patient was declared 47,XY,+i(18)(p10).

Imaging and testing

The patient underwent prolonged electroencephalogram (EEG) monitoring for 41-60 minutes. The recording during wakefulness contained 10 Hz alpha activity over the posterior head regions. There was bilateral temporo-occipital slowing greater on the right than the left, which is sharply contoured, with occasional right rhythmic theta accentuated during drowsiness. These results indicate a potential structural abnormality. No additional abnormalities occurred during hyperventilation or with photic stimulation.

**Figure 1:**
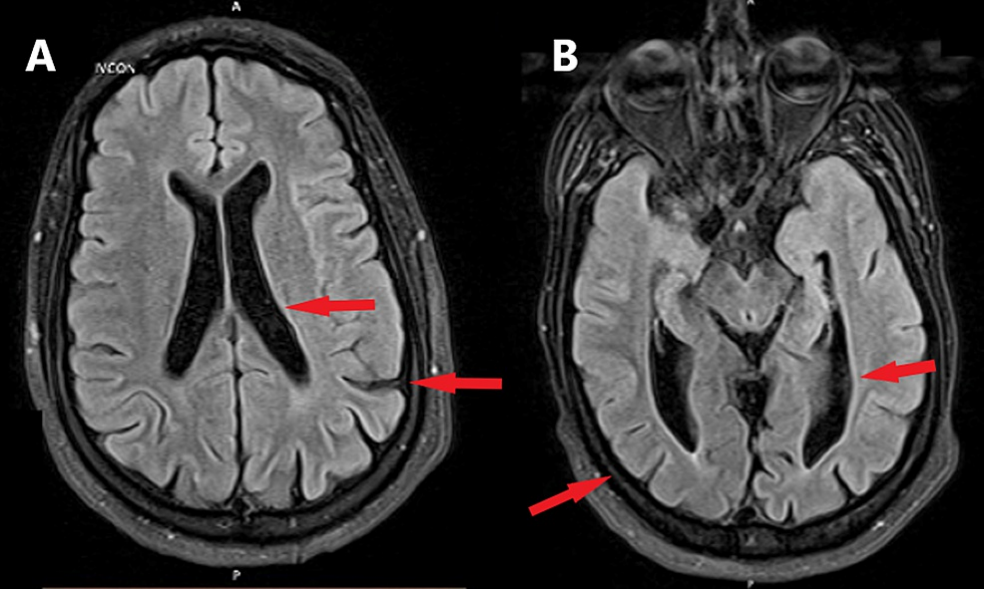
Axial brain MRI showing ventricular enlargement and diffuse cerebral volume loss most prominent in the bilateral parietal regions visible in both images (A,B) MRI - magnetic resonance imaging

A brain MRI without contrast revealed diffuse cerebral volume loss, most prominent in bilateral parietal regions, with associated ventriculomegaly (Figure 1). The paucity of white matter, more so in the posterior periventricular regions with subtle gliosis, was also seen, suggestive of periventricular leukomalacia. No acute or subacute cerebral infarction was noted.

On echocardiogram, no congenital defects were noted.

## Discussion

Tetrasomy 18p is a very rare medical condition affecting about one in 140,000 live births [2]. This case is suspected to be a de novo mutation, as most cases are; however, we were not able to perform a chromosomal analysis in the mother to confirm the origin of the supernumerary 18p isochromosome in our patient [1,3]. Tetrasomy 18p has been associated with various characteristic features, including low birth weight, muscle tone abnormalities, low set or dysmorphic ears, skeletal abnormalities, abnormal muscle tone, developmental delay, and mental retardation [4]. In the present report, our patient was born with low birth weight and was diagnosed with cerebral palsy as a child, which likely was a misdiagnosis and a manifestation of the tetrasomy 18 syndrome. Muscle tone abnormalities, especially spasticity, as is seen in our patient, have been reported in the literature [7,8]. Our patient also had scoliosis and kyphosis, which are the two most commonly reported orthopedic abnormalities in this syndrome. Various genitourinary abnormalities have been reported with tetrasomy 18p, including hypospadias and congenital malformations of the kidneys [4]. Although our patient suffers from chronic kidney disease, there are no structural abnormalities found on renal ultrasound. In a review of patients with tetrasomy 18p syndrome, Sebold et al. reported a few MRI findings showing enlargement of the lateral ventricles, thin or small corpus callosum, or signal abnormalities [4]. Our patient’s MRI revealed diffuse cerebral volume loss, most prominent in bilateral parietal regions with associated ventriculomegaly, which is similar to what has been reported in the literature. This patient suffers from hypertension, which is not a highly associated complication of tetrasomy 18p.

Findings associated with tetrasomy 18p span almost every system in the body. There have been reports of cardiac abnormalities, genitourinary malformations, gastrointestinal abnormalities, orthopedic abnormalities, and even stillbirth/early death. Therefore, patients diagnosed with this condition must have a multidisciplinary approach to their care. Caring for these patients mostly includes preventing complications from their congenital malformations and managing chronic conditions. Patients with suspected tetrasomy 18p should undergo genetic evaluation, counseling, and parental chromosomal analysis. For patients such as ours who are cognitively impaired, caregiver counseling is important. Patients with suspected tetrasomy 18p also need extensive evaluation by multiple specialties, including ophthalmology, audiology, ENT, cardiology, orthopedics, and neurology [4]. Gastrointestinal and endocrine evaluations are also necessary in addition to renal ultrasound to evaluate for renal abnormalities. Our patient was referred to neurology, orthopedics, and cardiology. Difficulty with behavioral and emotional regulation including difficulty adapting to new and unexpected changes have been reported in the literature and in our patient [9]. Cognitive impairments are one of the most characteristic features of tetrasomy 18p, ranging from mild to profound intellectual disability. Our patient has been unable to learn how to read; however, he is able to perform some activities of daily living with some assistance. Misdiagnoses are likely when these symptoms are evaluated in part and not as a whole. When dealing with a patient with intellectual disabilities and additional functional impairments, genetic testing should be performed immediately to rule out any genetic disorders, especially when newborn history and data are unavailable.

## Conclusions

More research is necessary on the management and long-term outcomes of patients with tetrasomy 18p; much of the literature has been on children with the disease with not as many studies looking at the long-term complications associated with this condition. Based on our literature search, our patient may be the oldest patient reported in the literature to be diagnosed with tetrasomy 18p. In addition, our case highlights the importance of considering genetic syndromes in the case of multisystem abnormalities, especially in the absence of newborn history. Our patient was misdiagnosed at an early age, and this could have been avoided with thorough history-taking and a broader differential.
